# Electrochemical and quantum mechanical investigation of various small molecule organic compounds as corrosion inhibitors in mild steel

**DOI:** 10.1016/j.heliyon.2021.e07952

**Published:** 2021-09-07

**Authors:** Mary Stephanie S. Carranza, Yves Ira A. Reyes, Erick Christofer Gonzales, Danielle P. Arcon, Francisco C. Franco

**Affiliations:** Chemistry Department, De La Salle University, 2401 Taft Avenue, 0922 Manila, Philippines

**Keywords:** Corrosion inhibition, Organic inhibitors, Electrochemical measurements, DFT

## Abstract

The corrosion inhibition property of selected small organic compounds was investigated using electrochemical measurements, including potentiodynamic polarization (PDP), linear polarization resistance (LPR), electrochemical impedance spectroscopy (EIS), and density functional theory (DFT) calculations. The inhibition efficiency (*IE* %) of the inhibitor on mild steel (MS) in 1 M HCl was then determined. Results show that the presence of the inhibitors resulted in decreased corrosion current density (*I*_corr_) values and increased polarization resistance (*R*_p_). Furthermore, the use of higher concentrations of inhibitors led to an increased inhibition efficiency. Tafel slopes and shifts in the *E*_corr_ values suggested that the inhibitors tested are mixed-type inhibitors that form a protective layer on the surface of the substrate. Of the organic compound inhibitors tested, the inhibitor 4-ethylpyridine (EP) exhibited the highest *R*_p_ values and inhibition efficiency values from the PDP, LPR, and EIS analyses, respectively. DFT calculations showed negative adsorption energies and confirmed the chemisorption of the inhibitors allowing for the formation of a hydrophobic protective film against corrosion and correlations between the quantum chemical values and electrochemical data were demonstrated. The results show the influence of the presence of electronegative O, S, and N atoms, as well as the role of aromatic rings in the promotion of surface protection by preventing aggressive ionic species from binding onto MS.

## 1 Introduction

Corrosion of mild steel (MS) is a contending issue in the field of industrial cleaning, mining, chemical processing, petrochemical engineering, material refining, and more. In corrosive environments, metallic corrosion can result in economic losses as well as health and safety hazards. External corrosion is one of the leading causes of pipeline transportation failure of natural gases, chemicals, and oil, which can lead to damage amounting to US $2.5 trillion in value, which is 3.4% of the global GDP (2013) [1]. Pipeline damage is caused by the corrosive effects of the environment such as soil acidity, oxygen concentrations in the atmosphere/water, CO_2_ concentrations in brine solutions, environmental temperature, natural catalysts present (i.e., less active metals such as copper or lead), and salt concentrations [2]. The presence of salt can lead to corrosion through the increase in acidity when dissolved in water [3,4]. Although these factors occur naturally and exist homogeneously in nature, these parameters are accelerants in the corrosion of mild steel.

Corrosion is a consequence of the chemical process that involves the conversion of Fe^2+^ to more stable Fe^3+^ oxide forms as well as other atoms present at the surface of the alloy. The destructive physical change is a cumulation of charge-transfer processes that take place at the metal-aqueous solution interface where a cathodic and/or anodic reaction occurs. To address these concerns, inhibitors are added to aqueous solutions in small portions to protect the metals from corroding. Inorganic inhibitors have been used which have been found to include phosphate, chromate, and other heavy metals. These components are now being gradually restricted or banned by environmental regulatory bodies due to their toxicity to the environment and their difficult disposal [5,6]. In contrast, organic inhibitors have been continuously investigated due to their versatility in molecular design, effectiveness in wide temperature ranges, low toxicity, low cost, good solubility, cathodic protection, and potential in coating applications [7, 8, 9].

Organic inhibitors correlate their corrosion inhibition efficiency to their adsorptive abilities onto the steel surface [10]. Corrosion inhibition efficiency relies on the coating or specifically, interactions present between the inhibitor and the surface of the MS. With higher corrosion inhibition efficiency, corrosion is slowed, and the anodic/cathodic processes are intercepted. Adsorption depends largely on the structural and chemical characteristics of the organic compounds mainly; size, electron density distribution, and orbital behavior of interactive electrons [11]. The molecules can be adsorbed on the metal surface by four possible mechanisms; (a) formation of electrostatic bonds between the charged surface of the metal and that of the inhibitor, (b) interactions between the lone-pair(s) in the structure of the inhibitor molecule and the metal surface, (c) interaction of pi-electrons and the metal surface or (d) a combination of the three mechanisms mentioned [12, 13]. The presence of S, N, O, and P atoms have been found to demonstrate these patterns in their role in corrosion inhibition, especially when arranged in heterocyclic rings. A study tested highly substituted benzoic acid and thiocarbohydrazide rings and found that with increasing inhibitor concentration a direct correlation with inhibition efficiency (%) resulted [14]. Several studies have also tested inhibition efficiency by classes of compounds such as those of amino benzonitrile, pyrazine, and benzimidazole derivatives where the mentioned compounds attributed chemisorption for its effectiveness [15, 16, 17]. Literature review has also shown that the high electron density of sulfur, nitrogen, oxygen, and phosphorus together with the presence of double and triple bonds amplifies the adsorptive properties of the inhibitor [18, 19]. Quantum chemical calculations and molecular docking simulations have also been used to demonstrate the theoretical interactions between the inhibitor and substrate which has proven to be powerful in revealing the mechanism of inhibition [20, 21, 22, 23, 24, 25]. Although numerous studies have been done on the corrosion inhibition potential of synthetic organic compounds, this usually involved a relatively complex procedure for its preparation. Furthermore, the use of small organic compounds has been studied to infer its effectivity as a corrosion inhibitor and whether quantum chemical methods depicted any correlations to experimental results [26]. A previous study revealed that the application of DFT methods on small datasets led to inaccurate conclusions drawn from the quantum chemical values and trends [27]. Partial least squares (PLS) regression is a predictive technique used to correlate predictor variables and produce equations to model the outcome of a predetermined number of measurements [28]. In this study, experimental data was used to demonstrate correlations with quantum chemical values and ensures that the estimated regression factors have relevance towards the quantum chemical values obtained [29].

**Figure 1:**
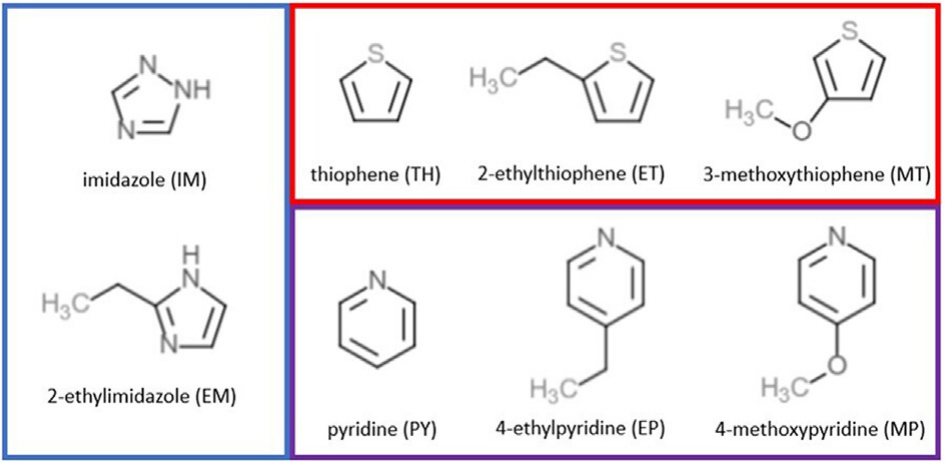
Structures of the small organic compound inhibitors in this study.

The corrosion inhibition effectiveness of selected small organic compound derivatives from parent compounds; imidazole, thiophene, and pyridine (Figure 1) were studied through electrochemical analyses and quantum mechanical calculations. Pre-determined concentrations of selected inhibitors were prepared in 1 M HCl solutions and analyzed using potentiodynamic polarization (PDP), linear polarization resistance (LPR), and electrochemical impedance spectroscopy (IES) measurements. DFT calculations on the isolated inhibitor molecules and Fe-inhibitor surface interactions were also carried out. The results of the calculations were then used to compare molecular structure, adsorption energy, and quantum parameters, to the corrosion inhibition ability of the organic compound inhibitors. Chemometrics (Partial Least Squares, PLS) was used to reveal correlations between experimental and theoretical findings.

## 2 Results and discussion

### 2.1 Potentiodynamic polarization (PDP) measurements

**Figure 2:**
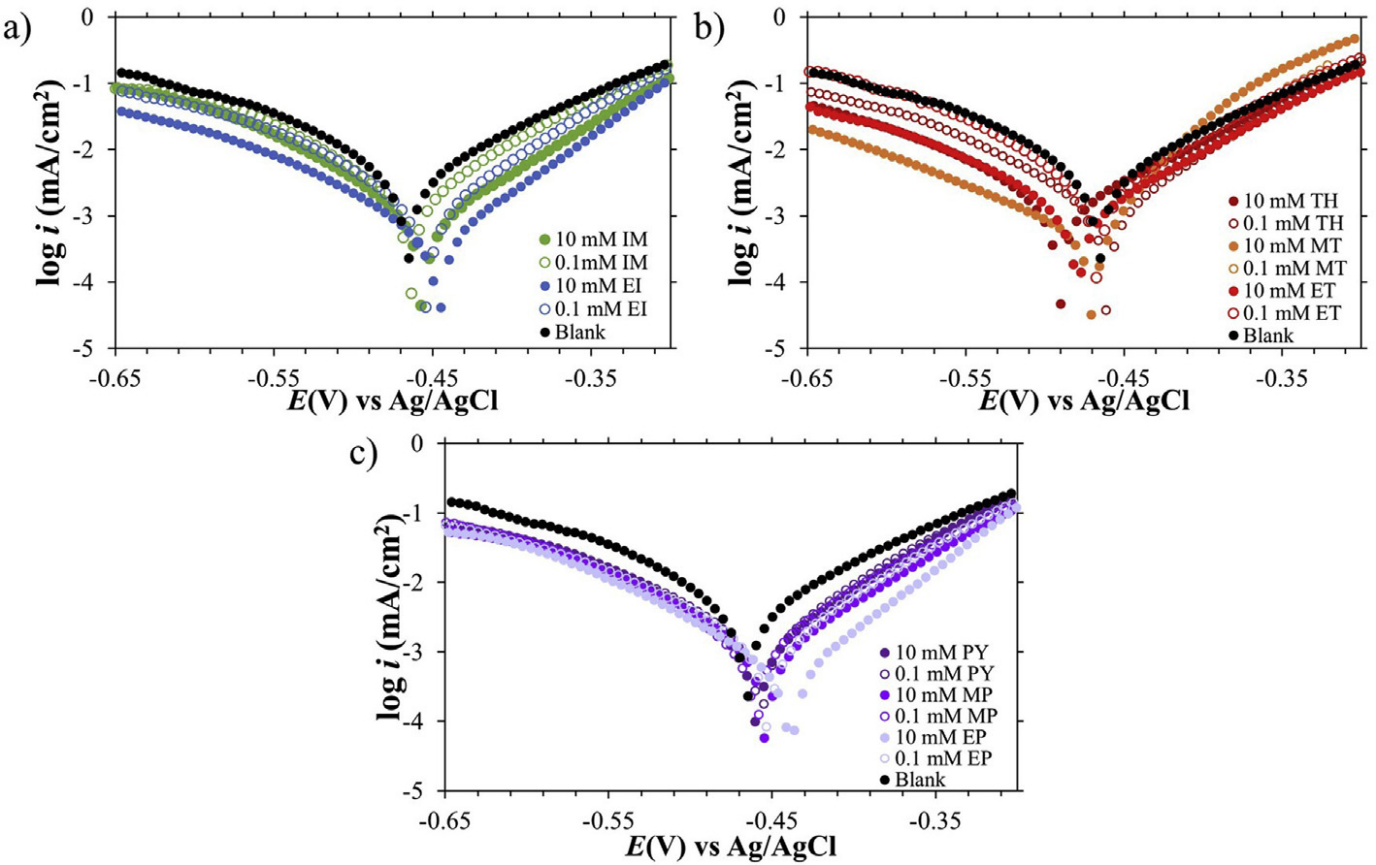
Polarization curves of corrosion in low-carbon steel samples in the presence of (a) imidazole and derivative-, (b) thiophene and derivative-, and (c) pyridine and derivative-inhibitors.

The potentiodynamic polarization results are depicted in Figure 2 and were collected after 20-minute immersions once a steady-state OCP was reached. This period was chosen based on the optimal conditions for the inhibitors to take effect and to collect viable data. Table 1 describes the electrochemical values extracted including the following: corrosion current (*I*_*corr*_), corrosion potential (*E*_*corr*_), open-circuit potential (*OCP*), anodic curve slope (*β*_*a*_), cathodic curve slope (*β*_*c*_), and inhibition efficiency (*IE %*). The corrosion inhibition efficiency was calculated from the equation:

(1) $$IE\;\%=\;\frac{i_{corr,o}-i_{corr,i}}{i_{corr,o}}\;\times 100\;\%$$

where *i*_*corr,o,*_ and *i*_*corr,i*_ are the corrosion current density of the MS in the HCl solution with no inhibitor, and in the HCl solution with inhibitor, respectively (equation 1). The mild steel samples were soaked in 1 M HCl solution at room temperature and were observed to exhibit higher inhibition efficiency values with larger concentrations of the inhibitor present. It also shows the corrosion inhibition effect of the organic compounds on both the anodic dissolution of metallic Fe and the cathodic production of hydrogen as seen from the lowering of the current density values Figure 2. Among the organic compounds, 10 mM EP exhibited the highest inhibition efficiency (85.9 %) with a corrosion current density of 3.657 μA/cm^2^, followed by 10 mM EI (IE% = 84.3, 4.092 μA/cm^2^) and 10 mM MT (IE % = 82.8, 4.464 μA/cm^2^).

**Table 1:** Polarization parameters and corrosion inhibition efficiency values of MS in the presence of the inhibitors in 1 M HCl.

	Conc (mM)	*I*_corr_ (μA/cm^2^)	-*E*_corr_ (mV)	*β*a (mV/dec)	-*β*c (mV/dec)	*IE*%
Blank		25.98	465.9	83.73	66.44	-
IM	0.1	13.66	464.0	70.99	65.57	47.4
	10	5.159	456.0	66.10	49.26	80.1
EI	0.1	11.14	457.3	69.14	70.14	57.1
	10	4.092	446.0	59.94	84.68	84.3
TH	0.1	9.571	490.5	63.58	77.48	22.1
	10	9.304	460.8	82.54	69.19	64.2
ET	0.1	18.88	467.0	73.86	68.54	27.6
	10	12.10	468.3	69.28	64.03	53.4
MT	0.1	11.27	498.6	82.73	71.69	56.6
	10	4.464	470.0	106.4	37.67	82.8
PY	0.1	11.41	457.1	64.35	81.70	56.1
	10	8.429	459.0	68.58	63.38	67.6
EP	0.1	10.81	452.4	66.23	93.76	58.4
	10	3.657	439.0	55.93	69.84	85.9
MP	0.1	8.959	459.9	69.10	72.81	65.5
	10	7.398	453.8	68.35	71.81	71.5

Organic compounds are generally considered as mixed-type inhibitors, while others are classified as cathodic or anodic inhibitors depending on their inherent molecular structure and properties [4,6,8,10]. Analysis of the extrapolated βc/βa Tafel slopes demonstrated that increasing concentration of the inhibitors did not cause any drastic changes in slope values (Table 1). This suggested that the inhibitors tested were mixed-type inhibitors and that the corrosion inhibition ability of the molecules involved the adsorption of the inhibitor onto the metal surface, reducing both the anodic and cathodic processes through the formation of a protective layer on the steel surface. These findings agree with published literature where minimal shifts in Tafel slope and *E*_corr_ values with the addition of mixed-type inhibitors Mt-3-PQPP (1-[3-(3-methoxyphenyl)-5-(quinoxa lin-6-yl)-4,5-dihydropyrazol-1-yl]propan-1-one) and Cl-4-PQPP (1-(3-(4-chlorophenyl)-5-(quinoxalin-6-yl)-4,5-dihydro-1H-pyra zol-1-yl)propan-1-one), were attributed to the adsorption of the molecules in a non-perturbative or weakly perturbative mode and geometrically optical coverage of the surface of MS [30]. Generally, the Tafel slopes were observed to increase as ethyl- and methoxy-substituents became more electronegative and concentrations increased, implying the impeding effect of the molecules in the anodic Fe dissolution and hydrogen reduction processes (Table 1).

### 2.2 Linear polarization resistance (LPR) measurements

LPR values depicted further the effect of the inhibitors on the corrosion of MS in 1 M HCl. Polarization resistance, *R*_p_ values were found to increase with the increase in concentration per organic compound inhibitor (Table 2). Corrosion inhibition efficiency from the LPR results was determined by the equation:

(2) $$IE\;\%=\;\frac{R\prime_{p}-R_{p}}{R\prime_{p}}\;\times 100,\;\%$$

where *R*_*p*_ and *R'*_*p*_ are the polarization resistance values with and without the addition of inhibitor to the 1M HCl solution, respectively (equation 2). The highest *R*_p_ values obtained for both concentrations was EP at 599.6 Ω cm^2^ (76.9 % IE) and 369.2 Ω cm^2^ (62.5 % IE) at 10 mM and 0.1 mM, respectively. This was followed by 10 mM PY (539.2 Ω cm^2^, 74.4 % IE) and 10 mM EI (538.7 Ω cm^2^, 74.3 % IE). This data is indicative of good inhibition character on mild steel similar to previously reported literature [31,32].

**Table 2:** Potentiodynamic polarization and inhibition efficiency values of MS in the presence of the inhibitors in 1 M HCl.

	Conc (mM)	*R*_p_ (Ω cm^2^)	*IE*%
Blank	-	138.3	-
IM	0.1	316.4	56.3
	10	467.2	70.4
EI	0.1	248.7	44.4
	10	538.7	74.3
TH	0.1	327.4	41.0
	10	245.9	59.9
ET	0.1	152.5	9.31
	10	178.1	22.3
MT	0.1	190.5	27.4
	10	330.2	58.1
PY	0.1	233.4	40.8
	10	539.2	74.4
EP	0.1	369.2	62.5
	10	599.6	76.9
MP	0.1	240.0	42.4
	10	314.6	56.0

### 2.3 Electrochemical impedance spectroscopy (EIS) measurements

**Figure 3:**
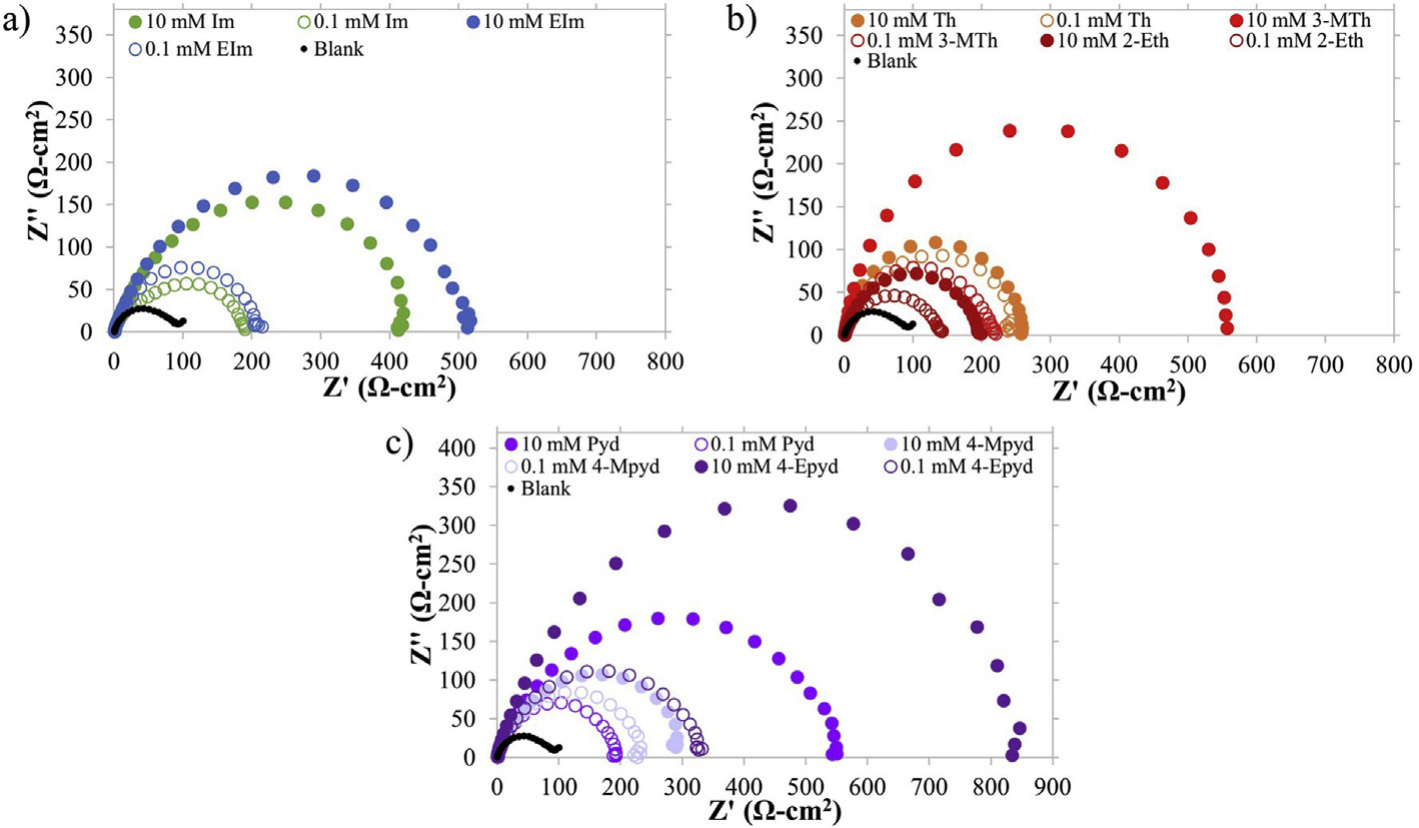
Nyquist plots of IM and derivatives, (b) TH and derivatives, and (c) PY and derivatives as inhibitors of mild steel corrosion in 1 M HCl.

Correlations between the selected organic compounds and their concentrations to electrochemical impedance measurements were made. The results were represented through the Nyquist diagrams demonstrated in Figure 3. An impulse with a known potential and frequency was applied to the system resulting in an impedance by the working electrode. This is primarily described by a series of points plotted using; real impedance (Z’), and imaginary impedance (Z”). The diameter of the semi-circle extrapolated in the Nyquist diagram represents the charge transfer resistance, alternatively called the charge transfer resistance *R*_ct_. The larger the diameter of the semi-circle, the higher the charge transfer resistance *R*_ct_ and the higher the inhibition efficiency. Eq. (3) describes the corrosion inhibition efficiency (*IE*%) calculated from the polarization resistance values with (*R*_ct,i_) and without (*R*_ct,o_) the inhibitors.

(3) $$IE\;\%=\;\frac{R_{ct,o}-R_{ct,i}}{R_{ct,o}}\;\times 100,\;\%$$

**Figure 4:**
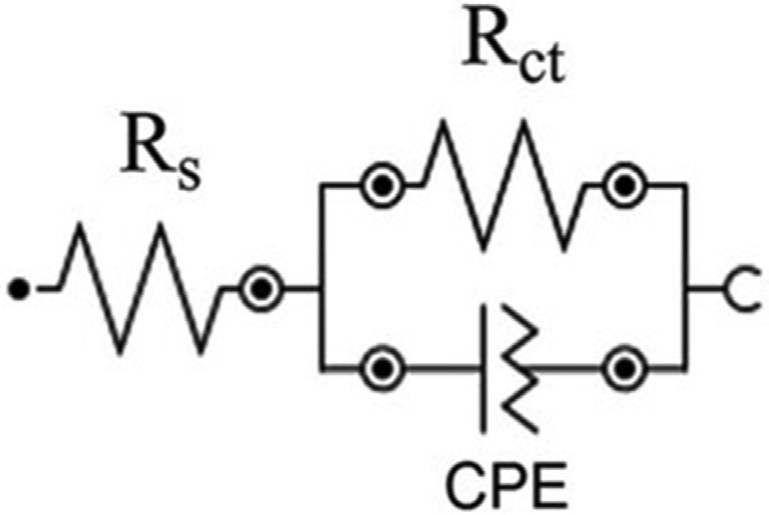
Fitting equivalent circuit for the EIS data analysis.

The accurate double-layer capacitance (*Z*_CPE_) values were determined by introducing the CPE in the fitting equivalent circuit (Figure 4) since the semi-circle diagrams are not perfect due to the surface irregularities. The impedance is therefore represented by the equation:

(4) $${\text{Z}}_{\text{CPE}}=\;\frac{1}{Y_{0}{\left(jw\right)}^{n}}\;$$

where *j* = $\sqrt{-1}$ , *w* is the angular frequency, *Y*_*0*_ and *n*, are the values of the CPE and exponent, respectively (equation 4). Correspondingly, the double-layer capacitance values (*C*_dl_) were determined using the following expression (equation 5):

(5) $$C_{dl}=\sqrt[{n}]{Y_{o}R_{ct}^{1-n}}$$

The Nyquist plots depict a typical semicircle attributed to elemental composition and distribution of the electrode surface, inhibitor adsorption, and the possibility of layer formation. Impedance values were observed to increase when the selected organic compounds were added as inhibitors. Plots with increasing concentrations of the inhibitors also demonstrated a direct correlation to the diameter and resulting *R*_p_ when compared to the blank control. Although both 10 mM IM and EI contain an aromatic ring with a N atom, 10 mM EI was found to exhibit an *R*_p_ of 519.8 Ω cm^2^ with 81.1% inhibition efficiency, greater than the inhibition efficiency of the former (at 78.0% efficiency, *R*_p_ of 411.0 Ω cm^2^) implying the role of the alkyl substituent in the adsorption of the inhibitor. A similar observation was made between ET and MT with the presence of an alkyl versus methoxy substituent to the parent compound; thiophene. Out of the total of eight compounds, 10 mM EP exhibited the highest *R*_p_ at 835.9 Ω cm^2^ (88.7% inhibition efficiency) which may be due to the orientation of the hydrocarbon chains facing towards the solution restricting the approach of aggressive ionic species onto the metal surface. Ethyl-substituent organic compounds were observed to have good inhibition capability by increasing the hydrophobicity of the surface of the MS sample. This concept was also demonstrated in another recent work where the effect of the length of the hydrophobic chain was studied in quinoline derivatives’ ability to impede corrosion [33]. Followed by the ethyl-substituent derivatives were the methoxy-containing derivatives at 82.6% (540.3 Ω cm^2^) and 67.5% (289.9 Ω cm^2^) for 10 mM MT and MP, respectively. This may be due to the presence of the aromatic ring and electron donor, O atom which would allow for the increased adsorption of the inhibitor onto the surface of the metal electrode.

A decrease in *C*_*dl*_ indicates a decrease in the local dielectric constant or an increase in thickness of the electrical double layer. This would also suggest that the inhibitors were successfully adsorbed onto the surface of the MS demonstrated by the charge transfer resistance value in the *C*_*dl*_ formula (equation 5). Similarly, *R*_*ct*_ values would also account for diffusion layer resistance, film resistance, and polarization resistance; denoting for the accumulation of corrosive products over the surface [34]. Based on the values in Table 3, all double-layer capacitance values of the inhibitors proved to be lower than that of the blank, suggesting the adsorption of the inhibitor molecules onto the surface of the MS samples. More specifically, PY, MT, and TH demonstrated the lowest *C*_*dl*_ values at 0.248 μF cm^−2^, 0.263 μF cm^−2^, and 0.318 μF cm^−2^, respectively. A variety of variables would influence this characteristic, including the size of the molecules, charge distribution, and/or ion-inhibitor interactions. As other electrochemical measurements implied, *C*_*dl*_ data reaffirms the anti-corrosive behavior of inhibitors used in 10 mM concentrations versus 0.1 mM concentrations.

**Table 3:** Impedance data and corrosion inhibition efficiency values for MS in the presence of inhibitors and 1 M HCl.

	Conc (mM)	*R*_p_ (Ω cm^2^)	*C*_dl_ (μF cm^−2^)	*IE*%
Blank	-	94.2	1.095	-
IM	0.1	189.8	1.088	50.8
	10	411.0	0.617	78.0
EI	0.1	209.3	0.671	55.0
	10	519.8	0.421	81.1
TH	0.1	249.6	0.580	62.3
	10	259.8	0.318	63.7
ET	0.1	140.8	0.427	33.1
	10	196.3	0.566	52.0
MT	0.1	217.8	0.441	56.7
	10	540.3	0.263	82.6
PY	0.1	194.5	0.530	51.6
	10	551.8	0.248	82.9
EP	0.1	336.8	0.470	72.0
	10	835.9	0.387	88.7
MP	0.1	237.2	0.595	60.3
	10	289.9	0.724	67.5

### 2.4 Theoretical calculations

**Figure 5:**
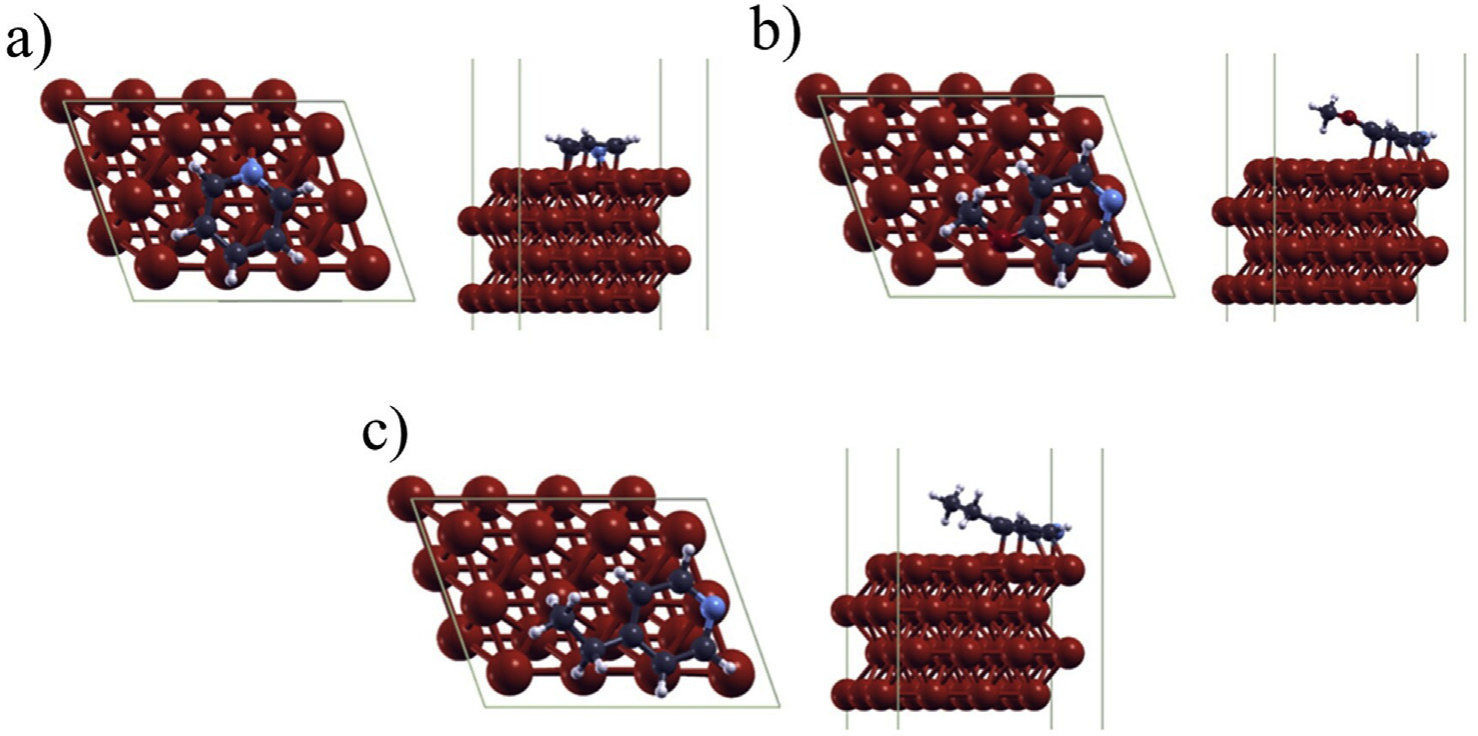
Optimized geometries of (a) PY, (b) MP, and (c) EP inhibitors on the Fe(110) surface.

The bonds formed between the inhibitors and the surface of the metal sample are attributed to the characteristic electronegative atoms present in the chemical structure as well as the orientation of the molecules with the unshared e's of the O and S atoms enhancing the inhibitory potential of the compounds [35]. The chemisorption of the inhibitors onto the surface of the metal sample allowed for the formation of a protective coating which prevented the onset of corrosion (Figure 5). Unlike that of 4-ethylpyridine, the hydrocarbon chains of 2-ethylthiophene or 2-ethylimidazole were oriented in closer proximity to the electronegative atom (Supplementary Information). This spatial distribution results in less surface area coverage and consequently less corrosion protection [36]. Among the three organic compound groups, pyridines and imidazole-based derivatives proved to be generally more efficient than their thiophene-based derivative counterparts which were found to be in good agreement with PDP and LPR data. Previous studies have shown that improved corrosion protection can be achieved by; (i) chain length, (ii) size of the molecule, (iii) bonding, aromatic/conjugate, (iv) strength of bonding to the substrate, (v) cross-linking ability, and (vi) solubility in the environment [37]. Correspondingly, the electrochemical data supports that the small organic compounds in this study had improved corrosion protection attributed to their molecular structure and interactions formed.

To explain the influence of the structure and interaction of the inhibitors onto the metal surface, theoretical studies were conducted using molecular and surface calculations. The DFT calculations were carried out to elucidate the following quantum parameters (Eqs. (6), (7), (8), (9), and (10)); the ionization potential (*I*), the electron affinity (*A*), the absolute electronegativity (χ), hardness (η), softness (σ), and the fraction of electrons transferred (Δ*N*):

(6) *I* = −*E*_HOMO_

(7) *A* = −*E*_LUMO_

(8) $$\text{χ}=\frac{I+A}{2}$$

(9) $$\text{η}=\frac{I-A}{2}$$

(10) $$\Delta N=\frac{\chi_{Fe}-\;\chi_{inh}}{2\left(\eta_{Fe}-\;\eta_{inh}\right)}$$

where χ_Fe_ and χ_inh_ are the absolute electronegativity of iron and inhibitor molecules, respectively, while η_Fe_ and η_inh_ represent the absolute hardness of iron and inhibitor molecules, respectively. The molecules' molecular orbitals and energies would have a direct impact on the strength of the bond made with the metal atoms. This adsorption phenomenon would be key to the corrosion mechanism and the effectivity of the organic compounds as inhibitors.

The results of the molecular calculations are consistent with the corrosion inhibition efficiency of the molecules. It can be observed that the positioning and orientation of the molecules on the surface of Fe results in differences in its corrosion inhibition efficiency (Figure 5). The more negative the adsorption energy an optimized inhibitor exhibits, the greater the tendency of the molecule to be adsorbed onto the surface of MS (equation 11). Electrochemical data in the previous sections showed the relatively high corrosion inhibition ability of EP, through the highly negative adsorption energy value obtained at -2.9031 eV. Following EP was PY and MP with adsorption energy values at -2.8009 eV and -2.6812 eV, respectively (Table 4). These findings confirm the possible corrosion inhibition mechanism of pyridine and its class of derivatives through the successful and efficient adsorption of the molecule onto the Fe surface layer. Figure 5 depicts the positioning of the inhibitor molecules and their possible interactions with the Fe surface. The π-electrons of the pyridine ring are found to form donor-acceptor interactions with the vacant *d*-orbital of the Fe atoms with the substituents orienting upwards towards the solution of H^+^ and Cl^−^ ions. The fraction of electrons, Δ*N* quantifies the transfer of electrons from the molecule to the metal, where electrons go from the less electronegative to the more densely packed Fe surface (equation 10) [38,39]. Although the magnitude of electron transfer for pyridine and its derivatives exhibited to be the lowest, this suggests an energetically favorable process where electrons from the heterocyclic ring are transferred unto the Fe atoms. Furthermore, Table 4 also shows high ionization potentials for the mentioned molecules, with *I*_PY_ = 7.43 eV, *I*_EP_ = 7.34 eV, and *I*_MP_ = 7.05 eV. In addition, these molecules exhibited the highest electronegativity values at *A*_PY_ = 1.13 eV, *A*_EP_ = 1.00 eV, and *A*_MP_ = 0.80 eV suggesting high corrosion inhibition potential. These theoretical results support the prediction that pyridine and its class of derivatives are relatively more efficient corrosion inhibitors compared to its imidazole and thiophene counterparts.

**Table 4:** Quantum chemical parameters of the solvated inhibitors and calculated adsorption energies.

Inhibitor	*I* (eV)	*A* (eV)	η	χ	σ	ΔN	*E*_ads_ (eV)
IM	6.59	-0.21	3.40	3.19	0.29	0.56	-2.03
EI	6.32	-0.10	3.21	3.11	0.31	0.61	-2.05
PY	7.43	1.13	3.15	4.28	0.32	0.43	-2.80
MP	7.05	0.80	3.13	3.92	0.32	0.49	-2.68
EP	7.34	1.00	3.17	4.17	0.32	0.45	-2.90
TH	6.68	0.72	2.98	3.70	0.34	0.55	-1.90
MT	6.06	0.69	2.68	3.38	0.37	0.68	-1.74
ET	6.37	0.66	2.86	3.52	0.35	0.61	-1.39

### 2.5 Statistical analysis

**Figure 6:**
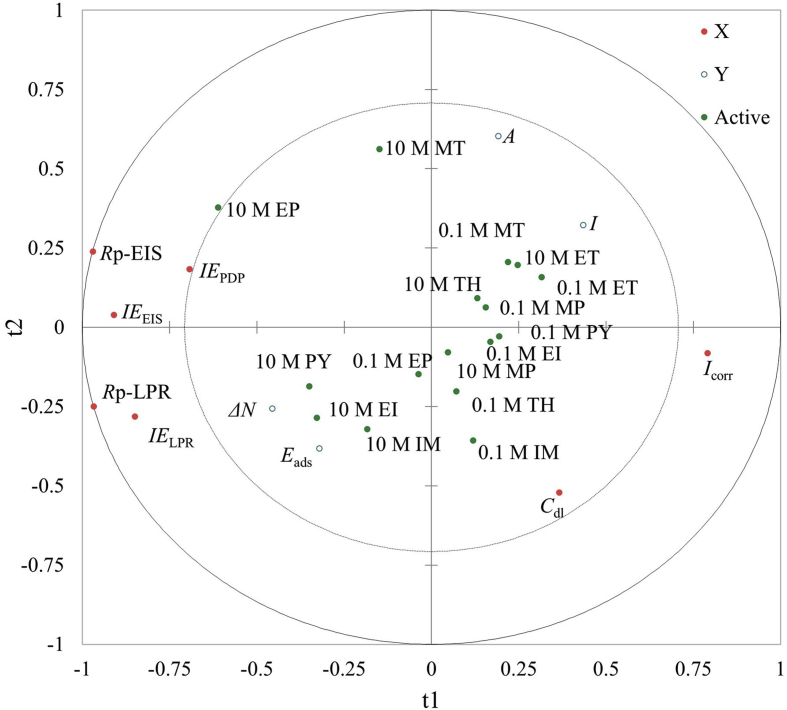
Partial Least Squares analysis biplot of variables X (electrochemical parameters) and Y (quantum chemical parameters) and observations (inhibitors and concentrations).

Multivariate partial least squares analysis was conducted to investigate the viability of the correlations made between electrochemical and quantum chemical data obtained for the chosen small organic compounds. Predictive value of the models was found to be low (Q^2^(cum) = 0.155 at 2 components). This value suggests that the quality of the fit would depend on the observations (inhibitors and concentrations involved). The cumulated R^2^Y and R^2^X(cum) that corresponds to the correlations between the explanatory (X) and dependent (Y) variables with the components were found to be low as well at 0.305 and 0.740 respectively (2 comp). This indicates that the electrochemical and quantum chemical variables alone do not account for the covariance observed between X and Y matrices. However, high correlations were found on the first two dimensions (Table 5). The correlation map demonstrated strong correlations found within the electrochemical parameters; *R*_*p*_*-*EIS, *IE*_EIS_, *IE*_PDP_, *R*_*p*_*-*LPR, and *IE*_LPR_. The inhibitor EP stands out showing strong correlation to polarization resistance and inhibition efficiency values. Three other clusters were derived: Δ*N* and *E*_ads_, *A* and *I*, as well as *C*_dl_ and *I*_corr_ values (Figure 6). These clusters depict strong correlations between the grouped variables with respect to the observations (inhibitors of 10 M and 0.1 M concentrations). In addition, a cluster of inhibitors are observed to correlate strongly to specific quantum chemical parameters: the electron transfer ability and resulting adsorption energies between the molecule and Fe(110) surface of MS when used in higher concentrations. At lower concentrations, the organic compound inhibitors show no relevant correlations to the variables tested.

**Table 5:** Correlation matrix of the variables with the *t* components.

Variable	*t*_1_	*t*_2_
*I*_corr_	0.791	-0.082
*IE*_PDP_	-0.694	0.184
*R*_p_-LPR	-0.968	-0.250
*IE*_LPR_	-0.850	-0.283
*R*_p_ - EIS	-0.971	0.239
*IE*_EIS_	-0.910	0.039
*C*_dl_	0.365	-0.521
*I* (eV)	0.434	0.322
*A* (eV)	0.191	0.604
Δ*N*	-0.457	-0.257
*E*_ads_ (eV)	-0.321	-0.383

∗PLS components (t1/t2), Q^2^(cum) = 0.035/0.155, R^2^X(cum) = 0.134/0.305, R^2^Y(cum) = 0.667/0.740.

## 3 Materials and methods

### 3.1 Preparation of inhibitors and steel substrates

Commercially available inhibitors (imidazole (IM), 2-ethylimidazole (EI), thiophene (TH), 3-methoxythiophene (MT), 2-ethylthiophene (ET), pyridine (PY), 4-ethylpyridine (EP), 4-methoxypyridine (MP)) were prepared in 0.01- and 10.0-mM concentrations by dissolving in 1 M HCl (Figure 1). The electrolyte solution of 1 M HCl was prepared from analytical grade 12 M (ACS reagent 37% Sigma-Aldrich) HCl.

Low-carbon mild steel (MS) samples were characterized using an optical emission spectrometer (OES) (Spectro Lab Lav M11). Table 6 describes the composition of the samples. The surface of the substrate was abraded using 240, 600, 1000, and 2000 grit emery papers. HPLC-grade acetone (≥99.9%, Sigma-Aldrich) was used to wash and degrease the mild steel samples followed by air-drying in a dry box before use.

**Table 6:** Elemental composition of the mild steel used in the study.

% C	% Si	% Mn	% P	% S	% Cr	% Mo	% Ni	% Cu	% Fe
0.0613	0.0107	0.2700	0.0140	0.0059	0.0343	0.0170	0.0247	0.0120	bal

### 3.2 Electrochemical methods

Electrochemical experiments were performed in a glass cell with a 100 mL test solution. The three-electrode system consisted of the counter electrode, a reference electrode with 3 M KCl electrolyte (Ag/AgCl), and the working electrode (WE). The WE was prepared using a cleaned 1 cm × 1 cm steel soldered onto the end of a copper wire, sealed with epoxy leaving one side exposed. Before the experiment, the test solutions were aerated with ambient air for at least 5 min. The electrochemical measurements were performed after the determination of the open-circuit potential (OCP) by letting the working electrode stabilize for 20 min. The potentiodynamic polarization (PDP) and linear polarization resistance (LPR) were carried out using Autolab PGSTAT128N at +/- 200 mV vs. open-circuit potential (OCP) at a scan rate of 0.5 mV/s and +/- 25 mV vs. OCP at a scan rate of 0.125 mV/s, respectively. The potentiostat was equipped with a FRA32M module accessory for the electro impedance spectroscopy (EIS) analysis. The measurement was done in 50 kHz to 0.1 Hz with 5 mV sine amplitude with respect to the OCP.

### 3.3 Computational methods

Quantum chemical calculations of the inhibitor molecules were carried out with Gaussian16 [40]. Geometric optimizations were done by using the B3LYP functional and 6-31+G(d,p) as the basis; with Grimme's D3 dispersion and BJ damping to account for the non-covalent interactions due to dispersion forces [41, 42, 43]. The solvent effects were considered by applying the polarizable continuum model (PCM) with water as the solvent [44]. Vibrational analysis was carried out to make sure there were no imaginary frequencies, and the optimized structures were at the minima. Energy calculations were also carried out at DFT/B3LYP-D3(BJ)/6-31+G (d,p).

Calculations involving the inhibitors and Fe surface were carried out with the PWSCF code of the Quantum-Espresso suite of programs [45,46]. The calculations were performed with the spin-polarized generalized gradient approximation density functional theory (DFT-GGA) of Perdew-Burke-Ernzerhof (PBE) using the plane-wave pseudo-potential method with ultra-soft pseudopotentials (USPP) [47,48]. The kinetic energy cutoff was set at 30 Ry and 300 Ry for the charge-density cutoff. The cold smearing method by Marzari-Vanderbilt with a smearing parameter of 0.02 Ry was used to account for the occupations [49]. For the Fe-inhibitor surface interactions, a slab model repeated under periodic boundary conditions was used to construct the Fe(110) surface. The supercell consisted of 3 × 4 surface unit cell and was composed of four iron layers with a ∼20 Å vacuum layer between the top of the ad-molecule and the adjacent slab. The bottom two layers were constrained to their bulk structure while the top two layers were allowed to relax. For the bulk Fe-bcc, the (8 × 8 × 8) k-point sampling mesh was used while the surface interactions were done at the gamma point. The adsorption energies *E*_ads_ of the inhibitors to the Fe surface were determined by the equation:

(11) *E*_ads_ = *E*_Fe-inh_ – *E*_Fe_ – *E*_inh_

where *E*_Fe-inh_ is the total energy of the Fe(110)-inhibitor, *E*_Fe_ is the total energy of the Fe110 surface, and the *E*_inh_ is the total energy of the inhibitor (equation 11).

### 3.4 Chemometrics

For statistical analysis, Excel's XLSTAT (Addinsoft version 2021.3.1.12345) was used to perform partial least squares regression. The number of components was determined based on the criterion and cross-validation results (method: Jackknife LOO).

## 4 Conclusions

The anticorrosion potential of MS in 1 M HCl solutions containing several small molecule organic compounds was evaluated using electrochemical measurements and quantum mechanical calculations. It was observed that higher inhibitor concentrations lead to an increase in the inhibition efficiency. From the Tafel slopes and corrosion potential values, the organic compounds in this study were determined to be mixed-type inhibitors. The corrosion process is inhibited by the adsorption of the inhibitors onto the Fe surface forming a protective layer. The molecular descriptors and negative adsorption energies have shown moderate correlations to the electrochemical results. This was supported by partial square analysis of clustering in correlation maps between electrochemical and quantum chemical variables. The aromatic ring of the inhibitors interact with the Fe surface via chemisorption and the alkyl chains were oriented towards the solution protecting the surface from the attack of the corrosive species, thus resulting in higher inhibition efficiency. This study is limited in thermodynamic studies and must be carried out to confirm the Langmuir isotherm characteristics of the chosen inhibitors. Surface microscopic spectroscopy must be done as well to verify the film-forming ability of the ethyl pyridine. Nonetheless, these findings suggest an environmentally friendly, organic, and accessible alternative to inorganic compound inhibitors.

## Declarations

### Author contribution statement

Francisco C. Franco Jr: Conceived and designed the experiments; Performed the experiments; Analyzed and interpreted the data; Contributed reagents, materials, analysis tools or data; Wrote the paper.

Mary Stephanie S. Carranza: Performed the experiments; Analyzed and interpreted the data; Contributed reagents, materials, analysis tools or data; Wrote the paper.

Erick Christofer Gonzales: Performed the experiments; Contributed reagents, materials, analysis tools or data.

Danielle P. Arcon: Contributed reagents, materials, analysis tools or data.

Yves Ira A. Reyes: Contributed reagents, materials, analysis tools or data.

### Funding statement

This study was supported by the 10.13039/100015405Department of Science and Technology - 10.13039/501100010982Philippine Council for Industry, Energy, and Emerging Technology Research and Development (DOST-PCIEERD), Philippines with Project No. 04628, 2018 and the University Research Coordination Office of 10.13039/100012938De La Salle University – Manila (DLSU-URCO), Philippines with Project No. 24 F U 1TAY18-1TAY19.

### Data availability statement

Data will be made available on request.

### Declaration of interests statement

The authors declare no conflict of interest.

### Additional information

No additional information is available for this paper.
