# Supplementary material for: Electrochemical and quantum mechanical investigation of various small molecule organic compounds as corrosion inhibitors in mild steel
Source: Heliyon. 2021 Sep 7;7(9):e07952. doi: 10.1016/j.heliyon.2021.e07952 (PMC8441079; doi:10.1016/j.heliyon.2021.e07952)
Supplement: Supplementary Information [file mmc1.pdf]

Supplementary Information

Electrochemical and Quantum Mechanical Investigation of Various Small Molecule Organic Compounds as Corrosion Inhibitors in Mild Steel

Mary Stephanie S. Carranza, Yves Ira A. Reyes, Erick Christofer Gonzales, Danielle P. Arcon, and Francisco C. Franco, Jr. *

Chemistry Department, De La Salle University, 2401 Taft Avenue, 0922 Manila, Philippines

Author to whom correspondence should be addressed; electronic email: francisco.franco@dlsu.edu.ph; tel: +63 2 85360230 (F.C. Franco Jr.)

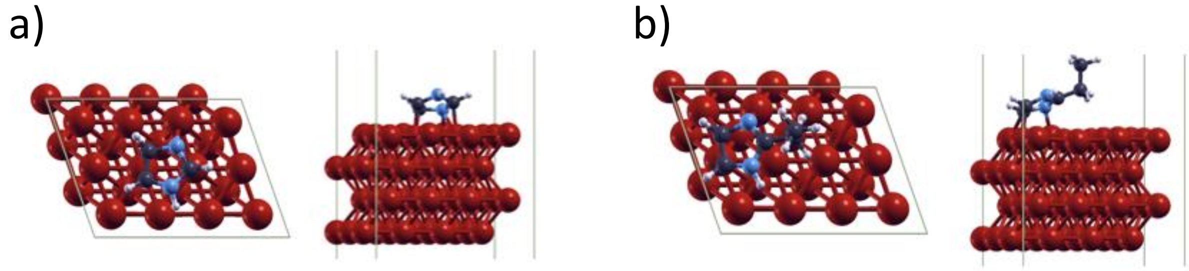

Figure S1. Optimized structures of (a) IM, and (b) EI inhibitors on the Fe(110) surface

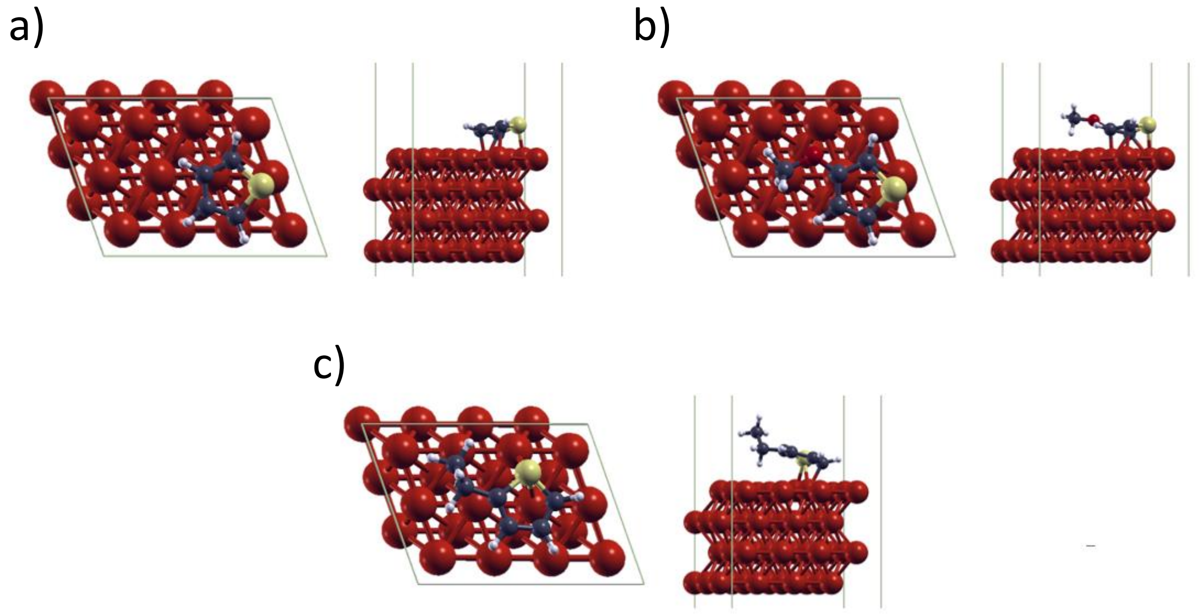

Figure S2. Optimized structures of (a) TH, (b) MT, and (c) ET inhibitors on the Fe(110) surface
